# Long-lasting changes in brain activation induced by a single REAC technology pulse in Wi-Fi bands. Randomized double-blind fMRI qualitative study

**DOI:** 10.1038/srep05668

**Published:** 2014-07-11

**Authors:** Salvatore Rinaldi, Marco Mura, Alessandro Castagna, Vania Fontani

**Affiliations:** 1 Department of regenerative medicine, Rinaldi Fontani Institute, Viale Belfiore 43, 50144 Florence, Italy; 2 Department of neuro psycho physical optimization, Rinaldi Fontani Institute, Viale Belfiore 43, 50144 Florence, Italy; 3 Institute of Radiology, University of Cagliari, 09042 Monserrato, Italy

## Abstract

The aim of this randomized double-blind study was to evaluate in healthy adult subjects, with functional magnetic resonance imaging (fMRI), long lasting changes in brain activation patterns following administration of a single, 250 milliseconds pulse emitted with radio-electric asymmetric conveyer (REAC) technology in the Wi-Fi bands. The REAC impulse was not administered during the scan, but after this, according to a protocol that has previously been demonstrated to be effective in improving motor control and postural balance, in healthy subjects and patients. The study was conducted on 33 healthy volunteers, performed with a 1.5 T unit while operating a motor block task involving cyclical and alternating flexion and extension of one leg. Subsequently subjects were randomly divided into a treatment and a sham treatment control group. Repeated fMRI examinations were performed following the administration of the REAC pulse or sham treatment. The Treated group showed cerebellar and ponto-mesencephalic activation components that disappeared in the second scan, while these activation components persisted in the Sham group. This study shows that a very weak signal, such as 250 milliseconds Wi-Fi pulse, administered with REAC technology, could lead to lasting effects on brain activity modification.

The Radio electric asymmetric conveyer (REAC) is a new technology for bio and neuro modulation. The REAC technology is based on the production of weak radiofrequency electromagnetic fields in the Wi-Fi bands (2.4–5.8 GHz) in order to generate a flow of electric micro currents in the body of the subject being treated. This current flow can be focused, as required, on specific areas through a probe-conveyor, peculiar of the REAC technology. In previous functional magnetic resonance imaging (fMRI) studies^1^,^2^ on healthy subjects, brain activity has been shown to be sensitive to modulation by REAC treatment administered according to a protocol named Neuro Postural Optimization (NPO), which has been proven to be effective in the correction of motor disorders (Fig. 1). In fact REAC-NPO is thought to modulate and optimize motor^2^ and postural strategies^3^. Also in patients with advanced Alzheimer's Disease^4^, REAC-NPO improved gait and number of steps × seconds. Cumulated experience derived from other studies supports the feasibility, safety and efficacy of this brain neuromodulation technique.

## Results

Two subjects, one of NPO-Treated group and one of Sham group, were excluded during statistical analysis due to the presence of artifacts that degraded the functional images. The average patterns of activation areas before treatment were similar in the NPO-Treated and Sham groups in that they were concordant with regard to the topography of the areas activated^5^. This similarity was expected given that the subjects in the two groups were performing an identical motor task. However, slight variations, statistically non-significant, between the groups with respect to the conspicuity and extent of activation were observed (Fig. 2a vs.2b). Compared to the group-averaged activation patterns before REAC-NPO or sham treatment, those recorded for the two groups after NPO-REAC or sham treatment showed a decrease in the magnitude of activation, a reduction in the conspicuity of the activated areas, and a disappearance of the thalamic activation component^6^,^7^ (Figs. 3a vs.3b). These changes were more marked in the NPO-treated group than in the Sham group. Furthermore, the cerebellar and ponto-mesencephalic activation components that were present in the NPO-Treated group pretreatment images (Figs. 4a), were completely absent in the NPO-treated post treatment images^7^. (Figs. 4b) This phenomenon was not observed when in the Sham group (Figs. 4c, d).

## Discussion

The present randomized double-blind fMRI qualitative study, although it was conducted on 33 patients, could still be considered as a preliminary study. We chose to perform a double-blind study in order to avoid any form of conditioning of the experimenters and of the subjects. Since it was important to verify that the results could be observed regardless of variables like sex or age, we chose a simple randomization instead of a stratified randomization sampling. Consequently we accepted the risk of a numerical imbalance of the group sizes, since the essential aim of the study was the intra-group comparison of the pre- and post-procedure fMRI results.

The clinical results from previous studies on healthy subjects^1^,^2^, neurological patients^3^ and subjects with advanced Alzheimer's disease^4^ seem to suggest the implementation of the same mechanism that we observe with fMRI after REAC-NPO to improve balance and gait. In this study, in both groups the post-treatment phase fMRI scans showed a reduction in the extension and in degree of activation in those areas activated during the leg motor task with respect to the first, pre-treatment phase scans.

As regards the sham group, one possible explanation of the decrease and change of activation in the parietal cortex and thalamus at the second scan, compared to the first scan, may be related to motor learning.

These reductions in activation were more pronounced in the REAC-NPO-treated group than in the Sham group. The most salient qualitative difference observed between the groups was that the REAC-NPO-treated group showed a disappearance of cerebellar and ponto-mesencephalic activation components following REAC-NPO, while these activation components persisted in the Sham group. These findings show that after REAC-NPO treatment, we obtain a reduction of the brain areas involved in the movement motor control. These results can be interpreted as a functional optimization of the brain structures that govern the coordination of motor control and balance. It is still unclear what are the mechanisms that make the effects of a single REAC-NPO treatment very stable over time, as verified in several clinical trials. Indeed, it is precisely this characteristic of long duration of the effect of REAC-NPO, which allowed us to carry out the administration of the treatment not during the fMRI scanning phase, as it happens in all other studies, conducted with different techniques^8^,^9^, but after this. Thanks precisely to the long duration of the effects of REAC-NPO, which greatly exceed the fMRI temporal resolution, we could then capture the effects of REAC-NPO treatment here showed. Probably the single REAC-NPO treatment could induce a neuromodulation of brain electrical activity and metabolism. Of course, as this study investigates a new phenomenology in the induction of neurological mechanisms in motor control and balance improvement, further studies will be needed.

## Methods

### Study cohort and study design

A total of 33 healthy, unpaid volunteers (19 females, 14 males), ranging in age from 19 to 54 years old (mean, 32.4 years) were recruited to participate in this double-blind randomized study. We chose to perform a double-blind study in order to avoid any form of conditioning of the experimenters and of the subjects, as the double blind tests are designed primarily to eliminate all the parameters conditioning the results.

We chose the simple randomization, since we believed the results should not be influenced by either the sex or age. We accepted the risk of a numerical imbalance of the group sizes, since the essential aim of the study was the intra-group comparison of the pre- and post-procedure fMRI results.

**Figure 1:**
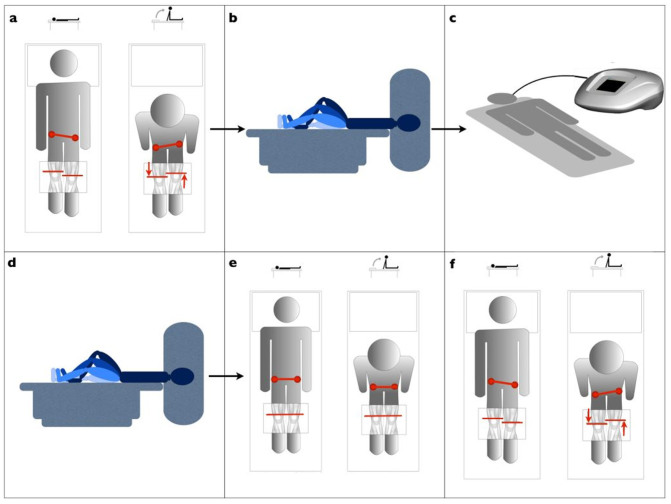
Study design overview. (a) Initial assessment of functional dysmetria (FD). (b) Pre REAC-NPO\Sham scans during performance of the motor task. (c) Administration of REAC-NPO\Sham. (d) Post REAC-NPO\Sham scans during performance of the motor task. (e) Post-REAC-NPO assessment of FD. (f) Post-Sham assessment of FD. A.C. prepared figure 1.

Before being subjected to the pre-treatment fMRI assessment, all subjects were investigated for the presence of functional dysmetria (Fig. 1a), so that at the end of fMRI examinations we could verify the clinical response to treatment in subjects treated compared to sham (Fig. 1e–f).

All subjects underwent a pre-treatment brain fMRI examination while performing a motor task already used in a previous study^2^ (Fig. 1b). After the pre-treatment fMRI assessment of all the patients enrolled, a staff member allocated each patient by coin-tossing randomization, assigning subjects to the REAC-NPO treatment (NPO-Treated) group or to the sham treatment control group (Sham). The randomization procedure placed 20 subjects (15 females, 5 males) in the NPO-Treated group and 13 subjects (4 females, 9 males) in the Sham group. Then a different staff member administered the therapy, unaware if he was performing the treatment or sham. Subjects belonging to the NPO-Treated group were treated with our previously validated NPO-REAC procedure2 (Fig. 1c), and Sham group subjects received a sham treatment. For the sham treatment, we followed the same procedures that were followed for the NPO-Treated subjects, except that the REAC device was not active. After the treatment phase, all subjects, after centering sequences, received a second fMRI examination while performing the same motor task (Fig. 1d). Neither the subjects nor the radiologist nor those who have examined the data were aware of which subjects were REAC treated and which ones were Sham treated. The timing between the REAC treatment and the post-treatment fMRI scan was 20 minutes.

This study was performed in accordance with the Helsinki Declaration. The work has been approved by the appropriate ethical committees related to the institution in which it was performed and subjects gave informed consent to the work. The clinical trial has been registered at Primary Registry Australian New Zealand Clinical Trials Registry with the Trials ID number ACTRN12611000366954 (Registration Title: Effect of a single REAC pulse on brain activity in healthy volunteers: fMRI double blind randomized pilot study).

### REAC technology and treatment

It is known that electromagnetic fields interacting with tissues can induce currents in them, which may also generate therapeutic effects. In neurological ambit, TMS is the most widely used technology^10^. TMS involves the administration to the scalp of a short (~200 ms) and powerful (0.2 to 4.0 T) magnetic pulse through a coil.

This magnetic pulse induces in the cortical surface below the coil an electric transient current, which causes the depolarization of the cell membranes and the transinaptic depolarization of the population of cortical neurons.

The effects of TMS single pulse on the neural activity are short-term, up to about 200 ms. TMS may also be administered with pulse trains. This mode of administration is called repetitive TMS (rTMS). The effects of rTMS persist longer than the single-pulse TMS, up to a few minutes^11^,^12^.

TMS and REAC technology have in common only the fact that both induce currents in brain tissue, but the differences between these technologies are substantial.

In fact, the operating diagram of the REAC technology can be summarized as follows:

The REAC technology generates an emission of microwaves (2.4 or 5.8 GHz) of very weak intensity (about 2 mW at the emitter). Solely thanks to the conveyer (asymmetric probe) of the REAC technology, this emission interacts with the biological tissues of the human body. This interaction generates, also in deep tissues, radiofrequency micro-currents, variables according to the molecular characteristics of the tissues. The sum of these radiofrequency micro-currents gives rise to a resulting micro current which, concentrated by the conveyer-probe of the device, exerts its therapeutic effect.

In the brain, the REAC technology has no limits in the depth of action, unlike TMS.

Previous works and extensive clinical experience show that a single pulse REAC-NPO determines long-term effects. The persistence of these effects can be easily controlled by the stability of correction of the functional dysmetria.

The REAC technology has two main fields of application, neuromodulation and biomodulation. In the context of neuromodulation specific REAC treatments have proven to be effective in treating various neuropsychiatric disorders^13^,^14^,^15^,^16^,^17^,^18^,^19^,^20^,^21^.

A REAC device (ASMED, Florence, Italy), model developed specifically for non-invasive brain stimulation, was used in this study. Subjects in the NPO-Treated group received a single 5.8 GHz REAC pulse that was 250 ms in duration. The REAC pulse was administered by applying the tip of the metallic REAC probe to a specific area of the ear located at the superior edge of the lower third of the scapha of the ear pavilion, according to the NPO protocol which is described in detail elsewhere^1^,^2^. At a distance of 150 cm from the emitter, the specific absorption rate (SAR) by the body is 7 μW/kg. The density of radio-electric current flowing to the subject during the single radiofrequency burst was 7 μA/cm^2^.

### Motor task

Given the physical constraints of fMRI examination, and according to the aim of evaluating the brain areas involved in the complex movements in walking, we used a motor task which simulates walking. The validity of this motor task has already been tested in a preliminary study^2^. The subjects performed a simple block design consisting of a 30-s-long knee movement task alternating with a 30-s-long rest, with the subject lying in a supine position (Fig. 1b and 1d). Each subject performed 30 motor task blocks during each scan. We specifically instructed the participants to execute the motor task in a slow but fluid motion such that the moving foot remained in contact with the MR scanner bed.

### Image acquisition, Image processing and statistical analysis

Because the purpose of this randomized double blind study is to verify the data obtained in the preliminary study^2^, we used the same procedures of image acquisition, image processing and statistical analysis.

Brain fMRI was performed with a 1.5 T unit (Philips Intera NT, The Netherlands), shown in Figure 1B. The survey was obtained with sequences of centering axial, sagittal, and coronal planes. Volumetric sequences with T1-weighted and T2-weighted gradient echo sequence were oriented upon these planes. The total duration of the fMRI acquisition, conducted while the subject performed the motor task, was about 40 minutes. Two radiologists who were not present for the REAC-NPO treatments performed the scans.

Acquired DICOM images were sent to a computer running on a LINUX operating system and then exported as compressed neuroimaging informatics technology initiative (NIFTI) files. The NIFTI files were then processed using fMRI expert analysis tool (FEAT) software in the functional MRI of the brain (FMRIB) software library (FSL). Two radiologists performed the first-level statistical analysis concurrently. Brain tissue was isolated from surrounding tissues using the Brain Extraction Tool (BET) in the FSL. The MCFLIRT Motion Correction tool was used for excluding “activations” caused by head movements.

The output was then processed with FMRIB's Improved Linear Modeling (FILM) tool in accordance with the block diagram of the study and taking into account the spatial parameters of the head motion detected by the MCFLIRT tool. A high-pass filter was used to adding the temporal derivative of the paradigm to the model. Spatial smoothing using a Gaussian kernel of FWHM 8.0 mm was applied. The results were registered with FMRIB's Linear Image Registration Tool (FLIRT).

Activation images were superimposed onto template image from the Montreal Neurological Institute provided with FSL (MNI152).

## Author Contributions

**Figure 2:**
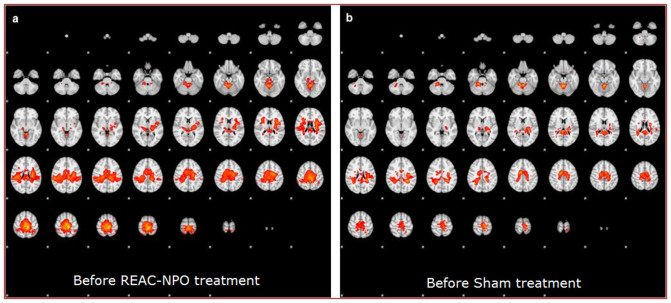
The average patterns of activation areas before treatment were similar in the NPO-Treated (a) and Sham (b) groups. Slight variations observed are statistically non-significant between the groups with respect to the conspicuity and extent of activation.

**Figure 3:**
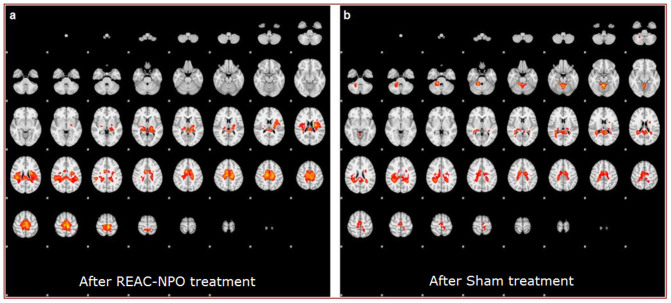
The average patterns of activation areas after REAC-NPO (a) or Sham treatment (b) showed a reduction in the extension and in degree of activation of the activated areas, respect to the first, pre-treatment phase scans. These reductions were more pronounced in the REAC-NPO-treated group (a) than in the Sham group (b).

**Figure 4:**
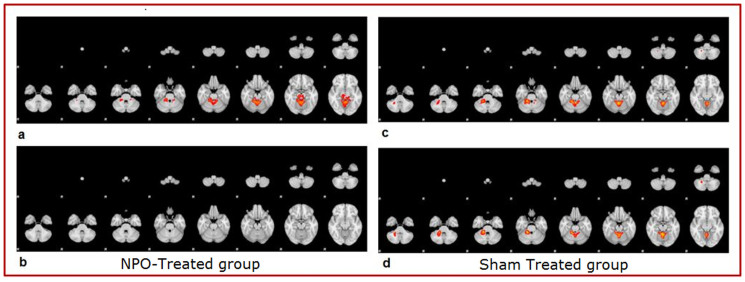
The cerebellar and ponto-mesencephalic activation components that were present in the NPO-Treated group pretreatment images (a) and in Sham group pretreatment images (c) were completely absent in the NPO-treated post treatment images (b). This phenomenon was not observed when in the Sham group (d).

S.R. and V.F. invented REAC, developed the experimental design and wrote the manuscript. M.M., conceived and executed most of the experimental plan, wrote the manuscript and prepared figures 2–4. A.C. performed the experiments and prepared figures 1. All authors reviewed the manuscript.
